# Assessment of disability and disease burden in neuromyelitis optica spectrum disorders in the CIRCLES Cohort

**DOI:** 10.1038/s41598-024-75013-z

**Published:** 2024-10-30

**Authors:** Shervin Gholizadeh, Alex Exuzides, Jennifer Sinnott, Chella Palmer, Michael Waltz, John W. Rose, Anna Marie Jolley, Jacinta M. Behne, Megan K. Behne, Terrence F. Blaschke, Terry J. Smith, Katelyn E. Lewis, Lawrence J. Cook, Michael R. Yeaman, on behalf of The Guthy-Jackson Charitable Foundation CIRCLES Study Group

**Affiliations:** 1 Genentech, Inc, South San Francisco, CA USA; 2 Department of Statistics, The Ohio State University, Columbus, OH USA; 3 University of Utah School of Medicine, Salt Lake City, UT USA; 4 The Guthy-Jackson Charitable Foundation, Beverly Hills, CA USA; 5 Departments of Medicine and Molecular Pharmacology, Stanford University School of Medicine, Stanford, CA USA; 6 University of Michigan Kellogg Eye Center, Ann Arbor, MI USA; 7 David Geffen School of Medicine, University of California, Los Angeles (UCLA), Los Angeles, CA USA; 8 Division of Molecular Medicine, Harbor-UCLA Medical Center, Torrance, CA USA; 9 Institute for Infection & Immunity, Lundquist Institute at Harbor-UCLA Medical Center, Torrance, CA USA

**Keywords:** Neuroimmunology, Neurodegeneration

## Abstract

Neuromyelitis optica spectrum disorders (NMOSD) comprise autoimmune diseases imposing substantial disability. We compared an NMOSD-targeted disability assessment of mobility, vision, and self-care domains (individually and composite) with the multiple sclerosis-targeted Expanded Disability Status Scale (EDSS) to assess NMOSD disease burden. An overall cohort (*n* = 505) and a subset of these patients with an enriched dataset (*n* = 198) were analyzed from the CIRCLES longitudinal, observational database of patients with AQP4-IgG–seropositive or –seronegative NMOSD in North America. Multinomial modeling was used to identify temporal correlates of disability improvement, stability, and worsening. Prior on-study relapse correlated with worsening mobility (OR, 3.08; 95% CI: 1.61–5.90), vision (OR, 3.99; 95% CI: 2.03–7.86), self-care disability (OR, 1.90; 95% CI: 1.07–3.38), and mean composite index disability (OR, 4.20; 95% CI: 1.71–10.34). Higher vision disability was associated with Black race, shorter time on-study, and AQP4-IgG–seropositive status in patients ≥ 18 years (*p* < 0.05). Disease onset phenotype and sex correlated with pain interference (*p* < 0.05). These correlates of NMOSD disability were undetected by EDSS. The CIRCLES real-world experience supports the need for NMOSD-specific disability assessment to improve recognition of disease burden, facilitate proactive clinical management, offer insights into resilience, and inform clinical trial design.

**Supplementary Information:**

The online version contains supplementary material available at 10.1038/s41598-024-75013-z.

## Introduction

Neuromyelitis optica spectrum disorders (NMOSD) comprise similarly presenting autoimmune diseases of the CNS^1,2^. Those affected may have significant disability due to inflammation, astrocytopathy, and demyelination mainly affecting the optic nerve(s) and spinal cord. Patients with NMOSD often experience relapses resulting in cumulative neurological disabilities such as vision loss and paralysis. The majority of these patients have detectable levels of serum antibodies targeting the water channel, aquaporin-4 (AQP4)^3^. The incidence of NMOSD is relative to geographical location, race, and ethnicity, where those of African and Asian descent are at a higher risk than other populations. Incidence estimates range from 0.05 to 10 per 100,000 people, have distinct geographical distributions, and indicate that females are disproportionately affected, at a ratio of up to 9:1 ^4,5^. Without treatment, NMOSD may result in blindness, irreversible loss of motor function and death^2^.

Traditional disability assessments in NMOSD employ methods adopted from other diseases, including multiple sclerosis, idiopathic optic neuritis, stroke, and aging (Table 1). The Expanded Disability Status Scale (EDSS) is often used in diagnosis, routine monitoring of disease status, and clinical trials, but it may be suboptimal for NMOSD. Originally created as the Disability Status Scale in 1954^6^, the EDSS was refined in 1983^7^ and has been extensively used since then, largely in clinical assessment of multiple sclerosis. EDSS relies on mobility as a heavily weighted core element, and this mobility is highly biased toward ambulation rather than upper-extremity function or fine motor coordination. Further, it uses a non-linear scoring scale, which limits its sensitivity in NMOSD. Scores ranging from 1 to 3 all reflect mild disability in a manner that is relatively insensitive to differences. Scores ranging from 3 to 6 bias activities of daily living but rely on relatively subjective interpretation. Finally, scores of 6 and higher almost exclusively focus on mobility. Of great relevance to NMOSD, EDSS is limited by suboptimal assessment of vision, bowel/bladder dysfunction, and self-care disability, and has little or no assessment of pain, cognitive impairment, or psychometrics regarding mental health. Additionally, there is a lack of area postrema assessment or other diencephalon relapses with EDSS. Each of these forms of disability can impose significant disease burdens and reductions in quality of life for patients with NMOSD. Thus, EDSS has key limitations regarding assessment of disabilities that are commonly seen in patients with NMOSD^8,9^. Other tools adopted for NMOSD include the Opticospinal Impairment Scale (OSIS)^3,10^, and the Modified Rankin Scale (MRS)^11,12^. These disability assessments are also adopted from measures for other diseases and are not designed to measure frequent clinical consequences of NMOSD. Thus, a significant unmet need persists for a disability assessment to detect disease burden and resilience specifically associated with NMOSD. Moreover, a disability assessment optimized for NMOSD and closely related conditions (e.g., myelin oligodendrocyte glycoprotein-antibody disease [MOGAD]) could be used to improve diagnostic accuracy, better monitor disease activity, quantify therapeutic effect, and enhance insights derived from clinical trials.

**Table 1:** Comparison of instruments historically used for disability assessment in NMOSD.

Rationale	NMOSD^26,27^	EDSS/FSS^7,28^	OSIS^10,29,30^	HAIMS^31^	MSFC^32–35^	MRS^12,36^	MBI^37,38^	IADL ^39,40^
**Instrument**	NMOSD Disability Domains and Index	Expanded Disability Status Scale/Functional Systems Score	Opticospinal Impairment Scale	Hauser Ambulation Index for MS	Multiple Sclerosis Functional Composite	Modified Rankin Scale	Modified Barthel Index	Instrument Activities of Daily Living
**Focus**	NMOSD	MS	ION/MS	MS	MS	Stroke	Stroke	Aging/AMS
**Features**	Based on NMOSD-specific real-world data	Accepted in routine clinical care and trials	Modified EDSS; no psychometric; not widely used	Similar to EDSS 10-point scale	Timed 25-ft Walk, 9-Hole Peg Test, Cognition Z Score	Global disability assessment; focus on walking	Rehabilitation bias 100-point scale	Focus on patients with mental illness
**Emphasis**	Mobility, vision, self-care (modular/index)	Ambulation FSS	Visual acuity, motor/sensor, and sphincter functions	Ambulation	Hand/leg/arm function and ambulation cognitive function	Non-specific 7-point scale	Ambulation, bowel and bladder control, hygiene/self-care	Routine activity (shop, chores, meals, finances, health care, etc.)
**Pain**	BPI	Minimal Assessment	–	–	–	–	–	–
**Cognition**	MoCA	Minimal Assessment	–	–	Limited Z Score	–	–	–
**Uncertainty**	e.g., MUIS^41^ [pending]	Minimal Assessment	–	–	–	–	–	–
**Relative test-retest ** **variability**	Not studied	High	Moderate	Low	High	Low	Low	Moderate
**Relative inter-rater** **agreement**	Not studied	High	High	Moderate	Low	Moderate	Moderate	Moderate

AMS, Aging Males’ Symptoms; BPI, Brief Pain Inventory; EDSS, Expanded Disability Status Scale; FSS, Functional Systems Score; HAIMS, Hauser Ambulation Index for MS; IADL, Instrument Activities of Daily Living; ION, idiopathic optic neuritis; MBI, Modified Barthel Index; MoCA, Montreal Cognitive Assessment; MRS, Modified Rankin Scale; MS, multiple sclerosis; MSFC, Multiple Sclerosis Functional Composite; MUIS, Mishel Uncertainty in Illness Scale; NMOSD, Neuromyelitis optica spectrum disorders; OSIS, Opticospinal Impairment Scale.

The Collaborative International Research in Clinical and Longitudinal Experience Study (CIRCLES) is a longitudinal, observational study of patients with NMOSD in North America. It collected clinical data and biospecimens from 2013 to 2020 ^13^. Its prospective aims included establishing a well-characterized cohort to advance understanding of NMOSD pathogenesis and epidemiology, improve clinical management, reduce disease burdens, and facilitate therapeutic development and eventual cures. The present study was designed to quantitatively compare disability assessment instruments used to evaluate patients with NMOSD. In this respect, the study aimed to assess the need for a disability assessment instrument optimized for patients with NMOSD, but not to propose such an instrument. Specifically, the study objectives were to (1) identify variables associated with disability change (worsening or improvement); (2) disclose potentially novel real-world correlates of NMOSD disability in CIRCLES patients; (3) compare traditional EDSS vs. NMOSD-specific mobility, vision, self-care, pain, and cognitive assessments using a modular and composite outcome strategy; and (4) contextualize the need for an NMOSD-specific disability and resilience assessment.

## Results

### Demographics and clinical characteristics

Relationships between variables and odds of disability change vs. stability were assessed in 505 patients with NMOSD, representing all patients included in the current study (Cohort 1). Of these patients, the majority were female (86.3%), White (55.0%), and AQP4-IgG seropositive (82.6%) (Table 2). The median age at first NMOSD episode was 38.7 years, and 29.3% of patients had ≥ 1 on-study relapse.

**Table 2:** Demographics and clinical characteristics of patients in CIRCLES Study cohorts 1 and 2.

Characteristic, *n* (%)	Cohort 1 (*N* = 505)	Cohort 2 (*N* = 198)
*Sex*		
Female	436 (86.3)	164 (82.8)
Male	69 (13.7)	34 (17.2)
*Race/ethnicity*		
Asian	47 (9.3)	22 (11.1)
Black or African American	124 (24.6)	49 (24.7)
Hispanic or Latino	56 (11.1)	24 (12.1)
White	278 (55.0)	99 (50.0)
Other	0	4 (2.0)
*Age at consent, years*		
< 18	14 (2.8)	6 (3.0)
18–34	101 (20.0)	38 (19.2)
35–48	162 (32.1)	59 (29.8)
49–64	171 (33.9)	75 (37.9)
≥ 65	57 (11.3)	20 (10.1)
*Age at first episode, years*		
< 18	42 (8.3)	18 (9.1)
18–34	154 (30.5)	59 (29.8)
35–48	177 (35.0)	72 (36.4)
49–64	113 (22.4)	40 (20.2)
≥ 65	19 (3.8)	9 (4.5)
*Follow up time on-study, years*		
< 2	168 (33.3)	56 (28.3)
2 to < 3	184 (36.4)	84 (42.4)
3 to < 4	78 (15.4)	21 (10.6)
≥ 4	75 (14.9)	37 (18.7)
Patient had a relapse on study	148 (29.3)	64 (32.3)
*AQP4-IgG serostatus*		
Positive	417 (82.6)	159 (80.3)
Negative	88 (17.4)	39 (19.7)
*Disease-onset phenotype*		
ON only and ON + brain	160 (31.7)	60 (30.3)
TM only and TM + brain	163 (32.3)	63 (31.8)
ON + TM and ON + TM + brain	55 (10.9)	17 (8.6)
Brain only	27 (5.3)	10 (5.1)
Confirmed through symptoms	72 (14.3)	22 (11.1)
Unknown	28 (5.5)	26 (13.1)
*Baseline ARR*		
< 0.5	325 (64.4)	144 (72.7)
0.5 to < 1	95 (18.8)	30 (15.2)
1 to < 1.5	34 (6.7)	12 (6.1)
1.5 to < 2	20 (4.0)	6 (3.0)
≥ 2	30 (5.9)	5 (2.5)
Unknown	1 (0.2)	1 (0.5)

AQP4-IgG, aquaporin-4 immunoglobulin G; ARR, annualized relapse rate; ON, optic neuritis; TM, transverse myelitis.

Cohort 2 consisted of 198 patients from Cohort 1 for whom an enriched dataset was available, including pain and cognitive assessment. The majority of patients in Cohort 2 were female (82.8%), White (50.0%), and AQP4-IgG seropositive (80.3%) and had a baseline annualized relapse rate (ARR) < 0.5 (72.7%; Table 2). At study consent, 37.9% of patients were 49 to 64 years old and 29.8% were 35 to 48 years old. Most patients experienced their first NMOSD episode between the ages of 35 and 48 years (36.4%) or 18 and 34 years (29.8%). The most common disease onset phenotypes for Cohort 2 included transverse myelitis or transverse myelitis plus brain syndrome (wherein lesions are identified as affecting one or more areas of the supratentorial brain regions, excluding the optic nerves, chiasm, and the diencephalon; 31.8%) and optic neuritis or optic neuritis plus brain syndrome (30.3%). Overall, 32.3% of patients experienced ≥ 1 on-study relapse.

### ***Change in disability metrics among all patients*** (Cohort 1; *n* = 505)

“No disability” emerged as the most common level of each first on-study disability metric at study consent (64.0%, no mobility disability; 47.9%, no vision disability; 45.7%, no self-care disability; 32.3%, no composite disability). Compared with vision and self-care, mobility disability scores were least likely to change during the study (71.3%, no change in mobility). However, changes in vision disability (54.7%) or self-care disability (46.9%) were considerably more frequent. Only 32.8% of patients reported no change in composite disability score.

### Variables associated with worsened or improved mobility

**Fig. 1:**
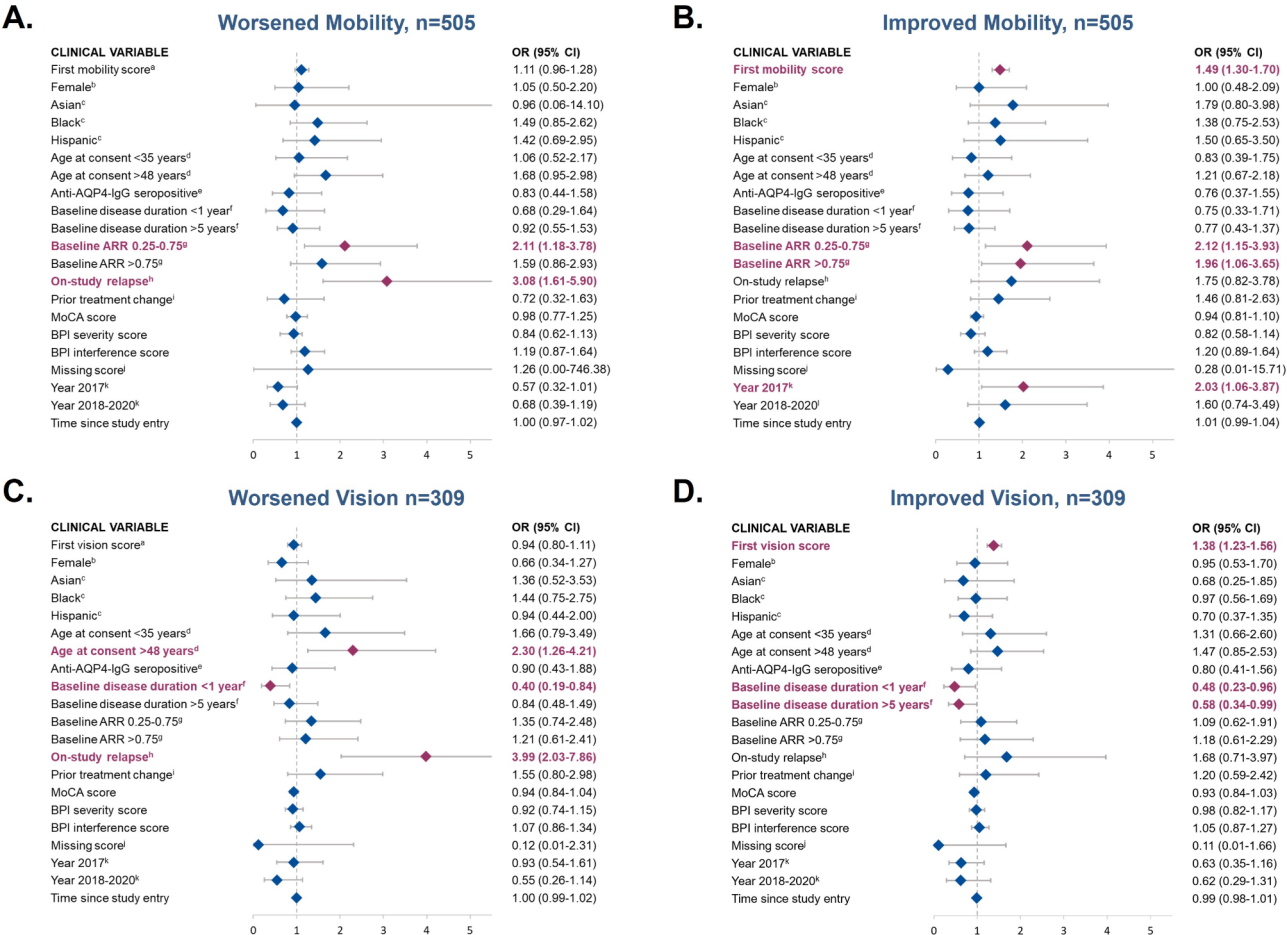
Assessment of clinical variables associated with worsened and improved mobility (panels **A** and **B**) or vision (panels **C** and **D**) compared with stability (neither improved nor worsened). Odds ratio (95% CI); red = significant association (*p*<0.05); blue = non-significant association (*p*>0.05). AQP4-IgG, aquaporin-4 immunoglobulin G; AAR, annualized relapse rate; BPI, Brief Pain Inventory; MoCA, Montreal Cognitive Assessment; OR, odds ratio. Panels A & B represent results from patients in Cohort 1 for whom mobility data were available. Panels C & D represent results from patients in Cohort 1 for whom vision data were available. ^a^Higher value of the score reflects worse disability. ^b^Reference, male; ^c^Reference, White. ^d^ Reference, age 35 to 48 years. ^e^ Reference, anti–AQP4-IgG seronegative. ^f^ Reference, 1 to 5 years. ^g^ Reference, ARR<0.25. ^h^ Reference, no prior on-study relapse. ^i^ Reference, no prior treatment change. ^j^ Binary variable indicating that MoCA, severity, and intensity scores are not available. ^k^ Reference, before 2017.

Baseline ARR of 0.25 to 0.75 vs. < 0.25 (OR, 2.11; 95% CI: 1.18–3.78) and on-study relapse (OR, 3.08; 95% CI: 1.61–5.90; Fig. 1A) were both significantly associated with worsened mobility during the study. A baseline ARR of 0.25 to 0.75 vs. < 0.25 (OR, 2.12; 95% CI: 1.15–3.93) and a baseline ARR of > 0.75 vs. < 0.25 (OR, 1.96; 95% CI: 1.06–3.65) were both significantly associated with a higher likelihood of experiencing improved mobility compared with remaining unchanged. An increase of 1 point in baseline mobility (i.e., worse baseline mobility) was associated with a higher likelihood of experiencing subsequent improved mobility, while controlling for all other factors (OR, 1.49; 95% CI: 1.30–1.70) (Fig. 1B).

### Variables associated with worsened or improved vision

Age > 48 as compared with age 35 to 48 years at study consent (OR, 2.30; 95% CI: 1.26–4.21) and experiencing an on-study relapse (OR, 3.99; 95% CI: 2.03–7.86) were associated with increased likelihood of worsening vision vs. remaining unchanged (Fig. 1C). Baseline disease duration of < 1 year vs. 1 to 5 years was associated with a decreased odds of worsening vision (OR, 0.40; 95% CI: 0.19–0.84). Similarly, a poorer baseline vision score (i.e., an increase of 1 point in baseline vision score) (OR, 1.38; 95% CI: 1.23–1.56) was significantly associated with higher likelihood of improving vision (Fig. 1D).

### Variables associated with worsened or improved self-care disability

**Fig. 2:**
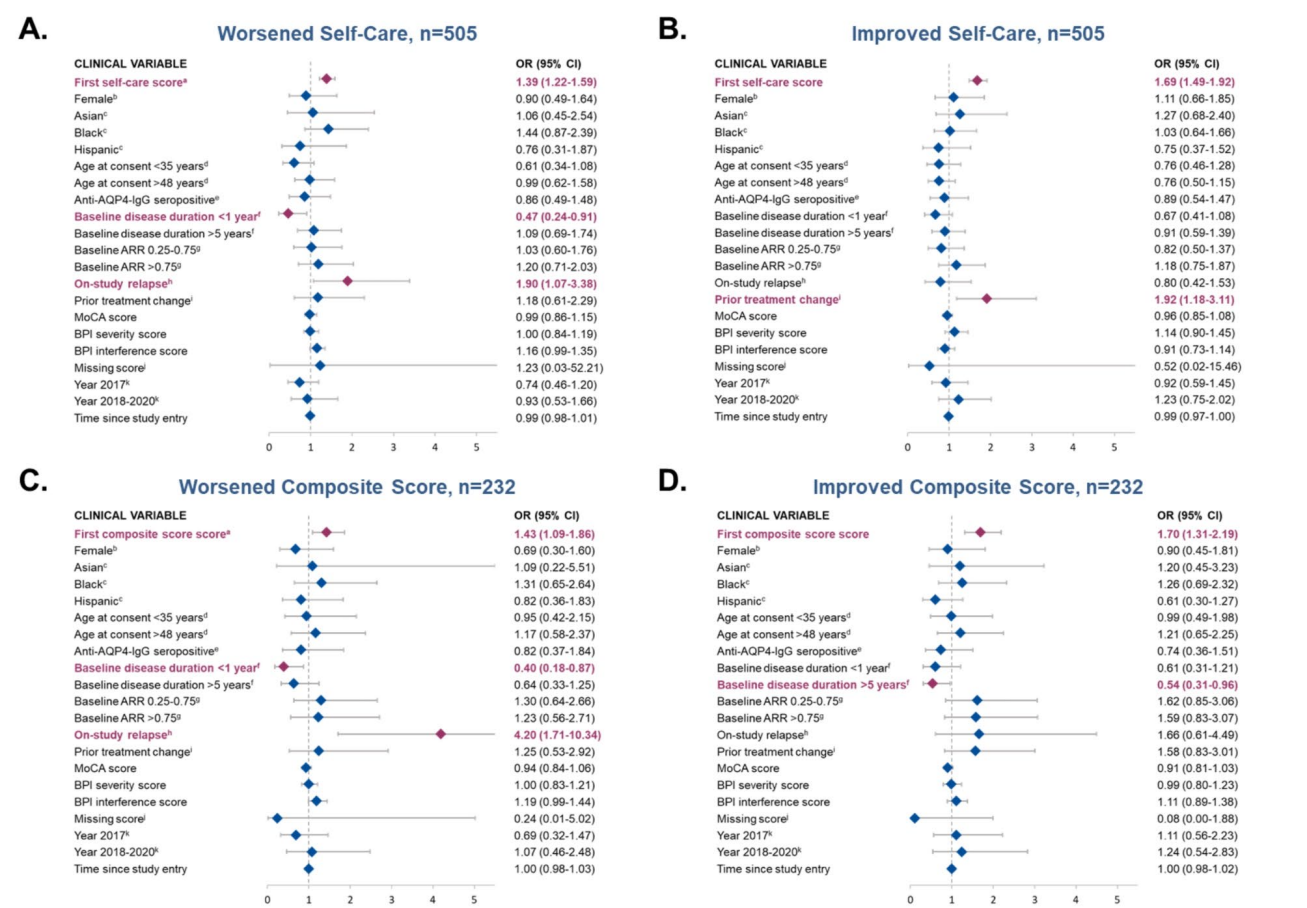
Assessment of clinical variables associated with self-care disability (panels A and B) or composite index score (panels C and D) as compared to stability (neither improved nor worsened). Odds ratio (95% CI); red = significant association (*p*<0.05); blue = non-significant association (*p*>0.05)AQP4-IgG, aquaporin-4 immunoglobulin G; AAR, annualized relapse rate; BPI, Brief Pain Inventory; MoCA, Montreal Cognitive Assessment; OR, odds ratio. Panels A & B represent results from patients in Cohort 1 for whom self-care data were available. Panels C & D represent results from patients in Cohort 1 for whom composite data were available. ^a^Higher value of the score reflect worse disability. ^b^Reference, male; ^c^Reference, White. ^d^ Reference, age 35 to 48 years. ^e^ Reference, anti-AQP4–IgG seronegative. ^f^ Reference, 1 to 5 years. ^g^ Reference, ARR<0.25. ^h^ Reference, no prior on-study relapse. ^i^ Reference, no prior treatment change. ^j^ Binary variable indicating that MoCA, severity and intensity scores are not available. ^k^ Reference, before 2017.

An increase of 1 point in baseline self-care disability score (i.e., worse baseline self-care) (OR, 1.39; 95% CI: 1.22–1.59) and on-study relapse (OR, 1.90; 95% CI: 1.07–3.38; Fig. 2A) were significantly associated with worsened self-care disability vs. no change in self-care ability. However, a poorer baseline self-care disability score (i.e., an increase of 1 point in baseline self-care disability score) (OR, 1.69; 95% CI: 1.49–1.92) and prior treatment change (OR, 1.92; 95% CI: 1.18–3.11) were significantly associated with a higher chance of improved self-care vs. no change in self-care ability (Fig. 2B).

### Variables associated with worsened or improved composite index score

An increase of 1 point in baseline composite score (i.e., a poorer score) (OR, 1.43; 95% CI: 1.09–1.86) and on-study relapse (OR, 4.20; 95% CI: 1.71–10.34) were both significantly associated with higher likelihood of worsening composite score (Fig. 2C). In contrast, an increase of 1 point in baseline composite score was also associated with a higher chance of experiencing a subsequent improvement in composite score (OR, 1.70; 95% CI: 1.31–2.19; Fig. 2D), whereas having a baseline disease duration of > 5 years vs. 1 to 5 years was associated with a reduced chance of an improving composite score (OR, 0.54; 95% CI: 0.31–0.96). Neither race or ethnicity nor AQP4-IgG serostatus were associated with disability change among individuals included in Cohort 1 of the current CIRCLES study (Figs. 1 and 2). Odds ratios for overall comparisons are summarized in Figs. 1 and 2.

### Correlates of NMOSD disability in enriched subset (Cohort 2; *n* = 198)

AQP4-IgG seropositivity in patients 18 years or older (i.e., those eligible for approved NMOSD therapeutics) exhibited a notable link between visual impairment, race or ethnicity, and the duration of time they were involved in the study (Table 3). Other comparative correlates of disability domain or composite scores relative to patient characteristics are summarized in Table 3.

**Table 3:** Correlates of disability domain or composite scores relative to patient characteristics in cohort 2.

Patient Characteristics, mean (SD)	Disability domains or composite indices (*N* = 198)							
	Mobility	Vision^a^	Self-Care	Composite^b^	EDSS^c^	MoCA^d^	BPI-Severity^e^	BPI-Interference^f^
*Sex*								
Male	0.85 (1.50)	0.79 (1.32)	0.76 (1.33)	0.86 (0.98)	3.44 (2.28)	23.29 (9.09)	1.76 (2.30)	1.59 (2.41)
Female	0.74 (1.39)	1.10 (1.60)	0.96 (1.41)	0.90 (1.21)	3.24 (1.98)	23.51 (8.54)	2.91 (2.58)	2.53 (2.83)
*p* value	0.682	0.237	0.436	0.846	0.636	0.901	0.013**	0.049*
*Age at onset, years*								
< 18	0.83 (1.72)	0.94 (1.63)	1.00 (1.78)	0.87 (1.43)	3.33 (2.36)	23.44 (8.83)	1.92 (2.26)	1.63 (2.45)
18–34	0.85 (1.35)	0.86 (1.47)	1.05 (1.55)	0.86 (1.06)	3.18 (2.18)	23.49 (9.05)	3.11 (2.95)	2.67 (3.14)
35–48	0.88 (1.55)	1.19 (1.63)	1.07 (1.27)	1.07 (1.26)	3.56 (2.04)	22.83 (9.31)	2.62 (2.52)	2.45 (2.89)
49–64	0.35 (1.08)	1.03 (1.51)	0.58 (1.22)	0.65 (1.11)	2.88 (1.63)	24.65 (6.48)	2.78 (2.27)	2.15 (2.24)
≥ 65	0.89 (1.05)	1.44 (1.74)	0.44 (1.01)	0.85 (0.78)	3.28 (1.89)	23.22 (9.22)	2.19 (1.78)	2.19 (2.37)
*p* value	0.376	0.716	0.300	0.507	0.537	0.888	0.455	0.679
*Age at consent, years*								
<18	0.83 (2.04)	0.33 (0.52)	0.83 (2.04)	0.61 (1.34)	2.92 (2.73)	21.33 (10.88)	0.38 (0.92)	0.17 (0.41)
18–34	0.61 (1.22)	0.66 (1.12)	0.84 (1.46)	0.68 (0.97)	2.61 (1.89)	25.55 (6.39)	2.65 (2.55)	2.63 (3.08)
35–48	0.51 (1.06)	0.98 (1.67)	0.83 (1.19)	0.76 (0.99)	3.02 (1.91)	23.10 (9.74)	2.71 (2.83)	2.37 (2.96)
49–64	0.95 (1.60)	1.24 (1.58)	1.09 (1.47)	1.08 (1.33)	3.71 (2.06)	23.00 (8.63)	2.81 (2.51)	2.19 (2.56)
≥ 65	1.05 (1.61)	1.50 (1.88)	0.80 (1.40)	1.08 (1.30)	3.80 (1.98)	23.00 (8.25)	3.21 (2.07)	3.24 (2.62)
*p* value	0.339	0.159	0.797	0.288	0.038**	0.565	0.209	0.173
*Race/Ethnicity*								
Asian	0.82 (1.56)	0.73 (1.52)	0.59 (1.22)	0.64 (1.18)	2.96 (2.28)	25.27 (6.36)	2.07 (2.13)	1.86 (2.35)
Black or African American	0.88 (1.47)	1.53 (1.72)	1.22 (1.48)	1.18 (1.34)	3.83 (1.94)	19.35 (10.90)	3.96 (2.61)	3.86 (3.26)
Hispanic or Latino	1.13 (1.73)	1.71 (1.88)	1.38 (1.50)	1.36 (1.37)	3.77 (2.08)	18.88 (11.57)	2.73 (2.96)	2.71 (3.24)
White	0.58 (1.19)	0.77 (1.32)	0.75 (1.35)	0.70 (0.98)	2.92 (1.94)	26.18 (5.35)	2.31 (2.36)	1.73 (2.22)
Other	1.25 (2.50)	–	1.00 (0.82)	0.75 (1.07)	4.13 (2.25)	24.50 (3.70)	1.06 (2.13)	0.72 (1.43)
*p* value	0.383	0.004**	0.104	0.032**	0.051*	< 0.001**	0.001**	< 0.001**
*AQP4-IgG serostatus*								
Positive	0.71 (1.37)	1.14 (1.63)	0.91 (1.41)	0.90 (1.22)	3.27 (1.98)	23.28 (8.84)	2.81 (2.55)	2.41 (2.75)
Negative	0.95 (1.52)	0.67 (1.13)	1.03 (1.35)	0.87 (0.97)	3.29 (2.24)	24.23 (7.68)	2.34 (2.61)	2.20 (2.93)
*p* value	0.376	0.035**	0.623	0.880	0.950	0.506	0.319	0.680
*Time on study*								
< 2 years	0.79 (1.44)	1.38 (1.86)	1.13 (1.36)	1.10 (1.26)	3.27 (2.27)	23.71 (8.79)	3.05 (2.61)	2.87 (2.99)
2 to < 3 years	0.75 (1.38)	1.18 (1.63)	1.01 (1.50)	0.95 (1.25)	3.39 (1.93)	22.69 (9.36)	2.74 (2.59)	2.38 (2.90)
3 to < 4 years	0.67 (1.43)	0.57 (0.81)	0.52 (1.03)	0.57 (0.80)	3.19 (2.11)	24.10 (6.47)	1.63 (1.77)	1.43 (1.81)
≥ 4 years	0.78 (1.46)	0.54 (0.93)	0.68 (1.33)	0.63 (0.94)	3.08 (1.90)	24.51 (7.75)	2.76 (2.73)	2.15 (2.56)
*p* value	0.989	0.029**	0.222	0.142	0.891	0.712	0.193	0.221
*On-study relapse*								
Yes	0.91 (1.56)	1.16 (1.72)	1.06 (1.40)	1.02 (1.29)	3.34 (1.85)	23.05 (9.29)	2.95 (2.79)	2.50 (2.91)
No	0.69 (1.32)	1.00 (1.48)	0.87 (1.39)	0.84 (1.10)	3.25 (2.11)	23.67 (8.30)	2.60 (2.45)	2.31 (2.73)
*p* value	0.333	0.533	0.356	0.341	0.762	0.648	0.397	0.665
*Baseline ARR*								
< 0.5	0.73 (1.37)	1.01 (1.59)	0.85 (1.40)	0.85 (1.18)	3.20 (2.07)	24.13 (8.13)	2.66 (2.55)	2.27 (2.74)
0.5 to < 1	0.73 (1.51)	1.33 (1.73)	1.33 (1.49)	1.13 (1.35)	3.33 (2.02)	20.67 (10.82)	2.76 (2.69)	2.60 (3.02)
1 to < 1.5	0.83 (1.34)	1.17 (1.19)	1.08 (1.31)	0.86 (0.85)	3.42 (1.83)	23.08 (8.22)	3.44 (2.25)	3.26 (2.96)
1.5 to < 2	–	1.17 (0.98)	–	0.39 (0.33)	3.08 (1.07)	21.50 (11.04)	1.88 (2.15)	1.00 (1.26)
≥ 2	1.60 (1.67)	0.40 (0.55)	1.40 (1.14)	1.13 (0.99)	4.10 (2.10)	25.60 (3.98)	3.80 (3.49)	3.91 (3.08)
*p* value	0.443	0.727	0.172	0.595	0.888	0.329	0.638	0.331
*Onset phenotype*								
(ON only) or (ON + brain)	0.47 (1.19)	1.10 (1.76)	0.63 (1.06)	0.74 (1.16)	3.22 (1.55)	23.47 (9.60)	1.95 (2.24)	1.64 (2.38)
(TM only) or (TM + brain)	0.87 (1.52)	0.95 (1.53)	1.03 (1.48)	0.89 (1.22)	3.26 (2.27)	24.37 (6.64)	2.79 (2.59)	2.48 (2.82)
(ON + TM) or (ON + TM + brain)	1.12 (1.62)	0.59 (0.87)	1.12 (1.27)	0.92 (0.85)	2.71 (1.96)	24.59 (6.68)	2.87 (2.73)	3.44 (3.30)
Brain only	0.20 (0.63)	0.50 (0.85)	0.10 (0.32)	0.27 (0.38)	2.55 (2.39)	27.70 (2.21)	2.28 (2.29)	0.96 (1.02)
Not confirmed clinically but through symptoms	1.14 (1.61)	1.09 (1.54)	1.32 (1.78)	1.23 (1.32)	3.84 (2.36)	22.32 (9.50)	3.76 (2.76)	2.90 (2.87)
*p* value	0.123	0.639	0.060*	0.233	0.376	0.472	0.052*	0.035**
*AQP4-IgG + and ≥ 18 years old*								
Yes	0.73 (1.39)	1.16 (1.65)	0.93 (1.42)	0.92 (1.22)	3.30 (1.99)	23.40 (8.74)	2.86 (2.55)	2.47 (2.76)
No	0.86 (1.47)	0.65 (1.09)	0.93 (1.32)	0.80 (0.95)	3.17 (2.20)	23.72 (8.25)	2.17 (2.55)	2.02 (2.85)
*p* value	0.602	0.018**	0.996	0.488	0.730	0.824	0.122	0.358

AQP4-IgG, aquaporin-4 immunoglobulin G; ARR, annualized relapse rate; EDSS, Expanded Disability Status Scale; MoCA, Montreal Cognitive Assessment.

*p < 0.1. **p < 0.05.

^a^White vs. Black or African American was significantly different (*p* = 0.035); <2 years of follow-up vs. ≥ 4 years of follow-up was significantly different (*p* = 0.053).

^b^No significant pairwise comparisons for race/ethnicity.

^c^Age 18–34 at consent vs. 49–64 was significantly different (*p* = 0.048).

^d^Asian vs. Black or African American (*p* = 0.036), White vs. Black or African American (*p* < 0.001), and White vs. Hispanic or Latino (*p* < 0.001) were significantly different.

^e^Asian vs. Black or African American (*p* = 0.027) and White vs. Black or African American (*p* = 0.001) were significantly different.

^f^Asian vs. Black or African American (*p* = 0.031) and White vs. Black or African American (*p* < 0.001) were significantly different. No significant pairwise comparisons for onset phenotype.

### Assessment of disability domains relative to clinical phenotypes (Cohort 2; *n* = 198*)*

In Cohort 2, higher vision disability scores were significantly associated with Black race than White race (mean [SD] vision score, 1.53 [1.72] vs. 0.77 [1.32]), AQP4-IgG seropositivity (1.14 [1.63] vs. 0.67 [1.13]), shorter time on study vs. longest (1.38 [1.86] for < 2 years on study vs. 0.54 [0.93] for ≥ 4 years), and AQP4-IgG–seropositive patients ≥ 18 years of age vs. those who are not (1.16 [1.65] vs. 0.65 [1.09]; Table 3). Importantly, the above variables were not associated with higher EDSS. Patients aged 49 to 64 years at study consent had higher mean (SD) EDSS scores than patients aged 18 to 34 years (3.71 [2.06] vs. 2.61 [1.89]); however, age was not associated with disability measured using any of the NMOSD-specific assessments. Age at disease onset, on-study relapse, and baseline ARR were all found to have non-significant relationships with each measure studied. In contrast, physical disability (composite, cognition, and pain severity/interference) was significantly associated with race or ethnicity (Table 3). In post hoc tests for multiple comparisons, Black patients had significantly worse mean (SD) Brief Pain Inventory (BPI) severity scores than Asian patients (3.96 [2.61]) vs. 2.07 [2.13]) or White patients (3.96 [2.61]) vs. 2.31 [2.36]). Likewise, Black patients had significantly worse BPI interference scores than Asian patients (3.86 [3.26]) vs. 1.86 [2.35]) or White patients (3.86 [3.26]) vs. 1.73 [2.22]). Black patients had significantly worse mean Montreal Cognitive Assessment (MoCA) scores than Asian patients (19.35 [10.90]) vs. 25.27 [6.36]) and White patients (19.35 [10.90]) vs. 26.18 [5.35]). White patients had significantly higher mean MoCA scores than Hispanic or Latino patients (26.18 [5.35] vs. 18.88 [11.57]). Pain interference significantly differed by disease onset phenotype. Female patients had significantly worse mean (SD) BPI severity and interference than male patients (2.91 [2.58] vs. 1.76 [2.30] and 2.53 [2.83] vs. 1.59 [2.41], respectively).

## Discussion

Real-world assessment of disability patterns unique to NMOSD would be an essential component of comprehensive care. Further, longitudinal assessment of such patterns may reveal underlying disease activity, enable prognostic capabilities for preventive intervention, and promote enhanced patient resilience and quality of life. However, such assessments are limited due to the fact that disability evaluation relies on measures and instruments that were not designed or optimized for NMOSD^8,9^. In the present study, assessment of modular functional domains and their composite index based on real-world patient experience identified limitations of traditional disability assessment in NMOSD. Most significantly, results revealed that demographic or clinical variables in NMOSD were primarily associated with visual impairment and a decline in the fine motor skills necessary for activities of daily living; these results were undetected or had poor sensitivity using EDSS. The current findings also offered insights into higher-resolution and domain-specific disability outcomes in NMOSD that were undetected by EDSS. Importantly, race or ethnicity appeared to be a unifying correlate of clinical disability, cognitive function, and pain in NMOSD. However, the quantitative relationships among these variables are non-identical (see Table 3 for specific correlation data comparisons among populations). Additionally, data suggest that the disease onset phenotype may have a greater effect on subsequent clinical disability, cognition, and pain than previously recognized^14^. Although older patients had a higher mean EDSS score than younger patients, age was not associated with disability using NMOSD-specific assessments, which suggests potential insufficient resolution power or over generalization in the EDSS instrument. Collectively, these findings provide substantive evidence supporting the need for development of novel instruments for improved detection of disability and resilience in patients with NMOSD.

Beyond bivariate analyses, multinomial models seek to identify variables associated with temporal worsening or improving disability compared with no on-study change in disability. This approach does not insist on the ordering of the outcomes (i.e., worsening, maintaining, improving), precisely because real-world associations may not match that ordering. For example, some patients with poor baseline self-care disability scores are more likely to worsen, perhaps because of more severe disease. In contrast, other patients are more likely to improve, perhaps because of enrollment proximate to relapse resolution, enhanced resilience, or other reasons. This outcome is unlikely to be an artifact due to regression to the mean, as time was included as a continuous variable in regression models. It is notable that this method yields some relationships that may appear counterintuitive; however, such outcomes are helpful in uncovering unforeseen correlates of change in disability status that warrant further investigation. Moreover, results that are consistent across all models indicate broader themes of disability that may be candidates as markers of disease activity or impact.

Another intentional strategy of the current investigation was to evaluate modular domains of disability in NMOSD independent of one another, compared with their integrated composite index. This rationale is supported by the fact that disability relative to distinct assessment modules such as mobility, vision, and self-care may or may not be linked. For example, it is reasonable to hypothesize that visual impairment may decrease mobility, given that spatial movement is based in part on visual cues^15,16^. By comparison, the ability to groom, study, or work may not be linked to mobility. Moreover, traditional disability assessments such as EDSS heavily weigh mobility based on ambulation over fine motor skills necessary for activities of daily living that are not dependent on ambulation^17^. As a result, disabilities distinct from mobility can be missed or diminished when combined into a composite index. Thus, the modular approach enabled dissection of disability focused on specific aspects of patient experience, as well as assessment of composite disability. Indeed, multinomial modeling of domain analyses performed in the present study revealed significant negative effects of relapse on change in disability in NMOSD, including those not detected by the composite index. For example, as hypothesized, experiencing a relapse on study was associated with worsening on all domains assessed. However, neither race or ethnicity nor AQP4-IgG serostatus were identified as primary correlates of disability change. This may be due to most correlations being based on EDSS, which has the aforementioned limitations. Age at study entry did not independently predict change in disability status, but older age was associated with worsening vision related to NMOSD. Although the prevalence of vision loss increases with age, with the highest prevalence in people aged 80 years and older^18^, in the present study, only a small proportion of patients in each cohort (~ 10%) were aged 65 years or older. Thus, the association between older age and worsening vision was unlikely to be related to age alone. Importantly, treatment change did not independently predict change in disability status but was associated with improvement in self-care score. This finding underscores the potential for high-efficacy therapies to reduce disease burden, improve patent quality of life, or both.

The current results reinforce the importance of preventing or minimizing relapse occurrence or severity in NMOSD relative to cumulative disability. New insights were also gained from the strategies used in the current analysis. For example, results revealed the relationship between pain or cognitive impairment and disability. In turn, these findings strongly support the need to better understand and address cognitive and pain issues through more-comprehensive assessment measures optimized for NMOSD. Importantly, the present study results support the hypothesis that outcomes in NMOSD are linked to race or ethnicity. For example, measures of pain and cognition were worse in Black patients and cognitive impairment was worse in Hispanic patients than in other study populations. Such results emerged even if clinical disease severity was equivalent. These results imply that healthcare disparities exist among patients with NMOSD, which need to be better recognized and addressed. Identifying and mitigating the potential causal factors imposing such disparate outcomes are urgent and are unmet needs in NMOSD and other areas of neurology^19^.

Such findings suggest new opportunities for understanding and measuring benefits of NMOSD therapeutics for their ability to reduce disease burden and/or enhance patient resilience. For example, in the era of high-efficacy therapy, the fact that treatment change was significantly associated with improved self-care may reveal clinical correlates of therapy that translate to reduced disease burden and greater quality of life. These measures support recent efforts to promote holistic resilience in patients with NMOSD through improved recognition and management of day-to-day symptoms in addition to long-term disease control^20^. The philosophy underpinning this approach is to treat the person, not just the disease.

Of special relevance, results from this study emphasize insights derived from assessing individual functional domains, compared with relying solely on composite indices in evaluating disability. For example, assessment of the vision module identified anti-AQP4 serostatus, temporal duration on study, and age in anti-AQP4–seropositive patients as correlates that were not detected in the NMOSD composite index or EDSS scores. This result is consistent with the independence of variables influencing disability in patients with NMOSD^21^. Likewise, the identified pain-specific correlates relative to sex and disease onset phenotype correlates with vision or pain were not detected in either the NMOSD composite index or EDSS assessments. It is possible that such findings reveal a basic limitation of combining disability measurements across activities that are not coupled or perhaps the presence of unintentional biases inherent to development of weighted scores. In any case, the present findings strongly support the hypothesis that the assessment of independent disability modules combined with the integrated composite score has the greatest chance of affording increased resolution and comprehensive themes in disability.

There are limitations to the current study design and analyses for consideration when interpreting the results. First, patient-reported outcomes have inherent limitations regarding subjectivity and non-standardized conditions of assessment. For example, improvements in self-care or mobility scores could reflect adaptive behaviors rather than improvement in the actual disease status. Likewise, the current study was not designed to control for age-related changes in vision as compared to general populations. Although such subjective aspects of data were controlled, they are not possible to eliminate. It is also possible that differences in available sample sizes for individual disability domains measured may have affected detection of statistical significance. Subclinical disease activity and non-clinical factors, including potential healthcare disparities, were not directly evaluated. However, as the results imply, it is reasonable to posit they could have influenced disability.

The present study was performed prior to the approval of therapies for NMOSD, and the impact of these therapies on disabilities is unknown. Future studies will be necessary to determine the effect of NMOSD therapies on correlates of disability or improved patient resilience. Moreover, patients newly diagnosed with NMOSD may have additional treatment options available and different treatment histories than those described here. It is important to qualify these results with respect to EDSS, as the current analyses were not designed to differentiate between EDSS overall, and its Functional Systems Score (FSS). We also note that while the present study was primarily designed to compare disability assessments, any future endeavors to establish an NMOSD-specific instrument will necessitate comparisons among different diseases to establish sensitivity and specificity, and for clinical validation. Finally, it should be emphasized that disability modules beyond mobility, vision, and self-care are likely to be essential to an eventual disability and resilience instrument optimized for NMOSD or related diseases. For example, addressing pain, cognitive impairment, bowel/bladder dysfunction, and psychosocial health are integral to comprehensive care for patients facing NMOSD. Assessment of diencephalon or area postrema syndrome–mediated disabilities present special challenges that will likely require additional cognitive assessment for comprehensive disability measures. Further, the disability domains presented herein would benefit from studies evaluating their relationship with patient-reported quality-of-life scores. These studies will allow better understanding of how changes in scores affect objective clinical vs. subjective patient perceptions of disease burden and quality of life.

Similar to many clinical trials, the study population in the present study did not fully reflect the race and ethnicity of patients who are most at risk of NMOSD, which may affect the generalizability of the results. It is vital that future studies assessing disability assessments in patients in NMOSD be representative of the populations most affected.

In summary, the current study provides quantitative evidence supporting the need for an NMOSD-specific disability assessment instrument. The methodologic approach and results offer new insights into the feasibility of developing a standardized and practical NMOSD disability and resilience assessment to address outcomes in real-world NMOSD, including self-care, pain, cognitive impairment, and psychological burden. Resilience resides in the difference between physical disability and perceived disability. By improving understanding of the physical burden of NMOSD, we reveal better ways to understand and promote resilience. Ideally, a future assessment of NMOSD burden will not only focus on disability but will also encompass resilience. A new instrument should also include both patient- and clinician-reported elements. Importantly, such an integrated instrument will ideally be applicable to anti-AQP4–seropositive disease and may potentially be applicable to MOGAD, seronegative NMOSD, and other closely related diseases. Furthermore, a new assessment of disability and resilience may need to be further specialized to be optimized for MOGAD and other closely related diseases individually. Uses of a new instrument may be applicable to gain insights into day-to-day management of symptoms, therapeutic effect, and the potential for disease progression independent of relapse, as well as strategies to improve resilience in NMOSD. Important to the potential development of such an assessment will be consensus regarding core data elements and comparative evaluation of clinical and patient-reported outcome data representing populations of patients with NMOSD throughout the world.

## Methods

### Patients and study design

Data from participants with diagnosed NMOSD enrolled in the CIRCLES study in North America were analyzed. CIRCLES is a prospective, real-world study that collected longitudinal clinical data and biospecimens from patients with NMOSD and relevant control participants from 15 academic medical centers in the United States and Canada^13^. Updated clinical history and biospecimens were obtained from 2013 to 2020 at 6-month intervals (Supplementary Resources 2 and 3). NMOSD was clinically diagnosed according to the Wingerchuk 2006 ^3^ or International Panel for NMOSD Diagnosis 2015^3 ^criteria and classified with respect to AQP4-IgG serostatus^13^. Nested eligibility criteria included NMOSD diagnosis, known serostatus (binary categorization of anti-AQP4 IgG positive or negative, where negative may include anti-myelin oligodendrocyte glycoprotein [anti-MOG IgG] positive serology, or serology negative for both of these autoantibodies ([i.e., seronegative NMOSD]), and willingness to participate in follow-up clinical visits at an interval of 6 months.

### Study cohorts

In the current longitudinal study, 2 cohorts of patients with NMOSD were analyzed. Assessment of change in disability was analyzed using Cohort 1 and assessment of disability domains was analyzed using Cohort 2, which was an enriched subset of Cohort 1 for patients who provided longitudinal data relative to cognitive function and pain and agreed to an extended clinical assessment period. Inclusion criteria for Cohort 1 consisted of an NMOSD diagnosis with ≥ 60 days’ follow-up and access to ≥ 2 disability scores in at least 1 of 3 functional domains (mobility, vision, and self-care). Inclusion criteria for Cohort 2 consisted of an NMOSD diagnosis, ≥ 60 days’ on-study follow-up, known AQP4-IgG serostatus, and scores from all 5 disability measures (mobility, vision, self-care, composite disability index, and EDSS).

### Disability domains

**Fig. 3:**
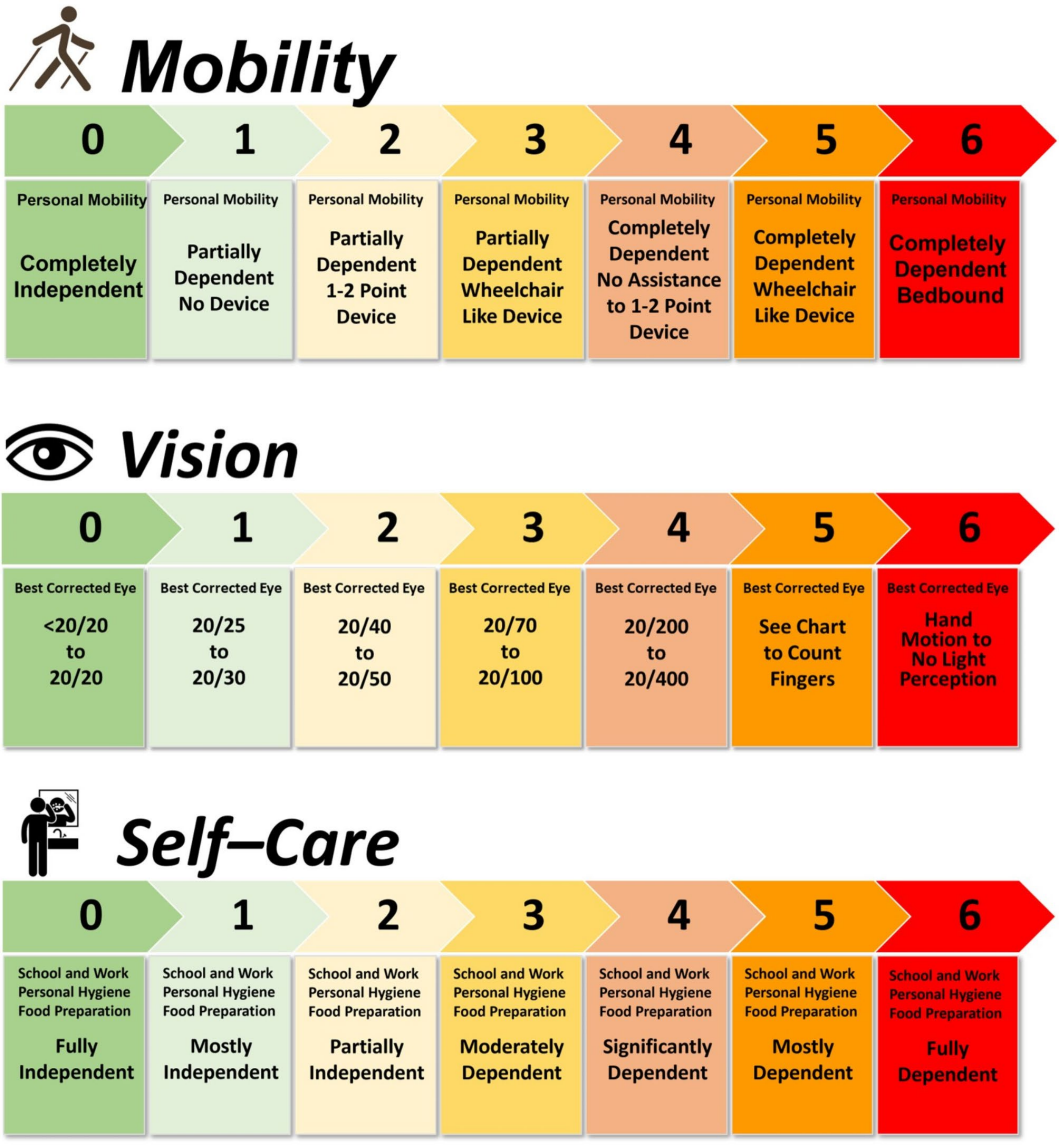
The 7-point disability scales in the vision (**A**),^a^ mobility (**B**),^b^ and self-care (**C**)^c^ domains, derived from patients with NMOSD.  NMOSD, neuromyelitis optica spectrum disorders.^a^ Visual acuity based on best corrected eye (corrective lenses, pinhole, or refraction). ^b^ Mobility scores reflecting wheelchair or equivalent assume impaired ability to transfer, ambulate, or both. ^c^ Self-care disability score derives from a cumulative 3-point score for each activity of daily life assessed as follows: attend school/work or perform routine housework; bathing/showering; bowel/bladder management; personal grooming; dressing; food preparation; eating. Each of these activities is independently scored as 0 = independent; 1 = partially dependent; 2 = completely dependent. Scores for each of these activities are summed for the overall self-care disability score; higher scores indicate greater disability.

A modular disability scale was developed to assess real-world disability in a manner that is not available with EDSS. Three disability domains representing a blend of clinician- and patient-reported outcomes were included: mobility, vision, and self-care. Information from an activities of daily living questionnaire and clinical neurological examination data (Supplementary Resource 4) were used to construct equally weighted 7-point scales (0 to 6) for each domain, where higher scores represented worsening disability (Fig. 3). To assess mobility disability, patient-reported levels of ability were mapped to disability regarding independence, use of assistance devices, or reliance on caregivers. The Snellen chart was used to measure standard visual acuity in the best-corrected eye during clinical neurological examination. Assessment of self-care was based on patient-reported information on abilities to perform bathing/showering, manage bowel/bladder functions, prepare meals, eat, dress, groom, and participate in school, employment, or housework. The average of the 3 individual domain scores was used to create the composite index score, provided that all 3 scores were collected and measured within 90 days of one another. An increase in disability score from baseline indicated worsening disability whereas a decrease from baseline indicated improvement in disability.

### Standard protocol approvals, registrations, and patient consents

All methods were carried out in accordance with relevant guidelines and regulations. A standardized protocol, manual of operations, patient study file, and informed consent or assent documents were approved by institutional review boards or respective clinical study sites. All study protocols were approved by the University of Utah/Data Coordinating Center Institutional Review Board, and/or by respective CIRCLES study site Institutional Review Boards. Written and verbal informed consent or assent was obtained before beginning the study procedures.

### Statistical analyses

The current study design allowed for continuous enrollment, and patients were assessed as they were enrolled. Data were obtained over the study period from 2013 to 2020. The longitudinal reference point was study enrollment date. Consistent with established clinical practices, the definition of relapse duration in the present study was 90 days^8,22–25^. On-study relapses were dated to their patient-reported onset date. Relapses beginning within 90 days before study consent were considered on-study relapses; those starting > 90 days prior to consent were classified as pre-study relapses.

Patients were assessed for disability in mobility, vision, and self-care domains and in a composite disability index that integrated these domains. In Cohort 1, a baseline-category logit model was used to identify variables that were temporally associated with disability improvement or worsening. Specifically, 2 logistic regression models were fit simultaneously to model the odds of improving vs. remaining unchanged or worsening when comparing pairs of consecutive disability assessments. Unchanging covariates included sex, race or ethnicity, age at study consent, AQP4-IgG serostatus, and disease duration at study consent, as well as first on-study mobility, vision, self-care, and composite scores. For the purposes of subset analysis, a composite of age > 18 years and AQP4-IgG –positive serostatus represented those patients eligible for FDA-approved therapies indicated for NMOSD. Each model included the most recent information for time-related covariates between pairs of consecutive disability assessments, including experiencing a relapse, treatment change, calendar year, time since study consent, MoCA score, BPI severity score, and BPI interference score. We also included a missing indicator (missing score) when neither MoCA nor BPI was (yet) available. Case-resampling bootstrapping was used to estimate standard errors accounting for within-individual correlation. In Cohort 2, differences in mean disability, cognition, and pain measurements were evaluated using *t*-tests with unpooled variance estimates for independent variables with 2 levels, or 1-way ANOVA for independent variables with 3 or more levels. These analyses tested correlations among mobility, vision, and self-care disability, disability measured by EDSS, as well as disabilities relating to cognition (MoCA), pain (BPI–severity or BPI–interference), and demographic or clinical variables. Post hoc analyses in the form of Tukey pairwise comparisons were conducted to identify specific differences between groups among significant ANOVA findings.

## Electronic supplementary material

Below is the link to the electronic supplementary material.

Supplementary Material 1

## Data Availability

CIRCLES data used in this study are available upon request to the corresponding author and subject to terms of agreement for their use.
