# Supplementary material for: Assessment of disability and disease burden in neuromyelitis optica spectrum disorders in the CIRCLES Cohort
Source: Sci Rep. 2024 Oct 30;14:26150. doi: 10.1038/s41598-024-75013-z (PMC11525583; doi:10.1038/s41598-024-75013-z)
Supplement: Supplementary file 1 — Supplementary Material 1 [file 41598_2024_75013_MOESM1_ESM.pdf]

Supplementary Resources

Assessment of Disability and Disease Burden in Neuromyelitis Optica Spectrum Disorders in the CIRCLES Cohort

Authors: Shervin Gholizadeh,[1†] Alex Exuzides,[1†] Jennifer Sinnott,[2,3] Chella Palmer,[3] Michael Waltz,[3] John W. Rose,[3] Anna Marie Jolley,[3] Jacinta M. Behne,[4] Megan K. Behne,[4] Terrence F. Blaschke,[5] Terry J. Smith,[6] Katelyn E. Lewis,[3] Lawrence J. Cook[3‡] and Michael R. Yeaman[7*‡] on behalf of The Guthy-Jackson Charitable Foundation CIRCLES Study Group[§]

Author affiliations: [1]Genentech, Inc., South San Francisco, CA, USA; [2]Department of Statistics, The Ohio State University, Columbus, OH, USA; [3]University of Utah School of Medicine, Salt Lake City, UT, USA; [4]The Guthy-Jackson Charitable Foundation, Beverly Hills, CA, USA; [5]Departments of Medicine and Molecular Pharmacology, Stanford University School of Medicine, Stanford, CA, USA; [6]University of Michigan Kellogg Eye Center, Ann Arbor, MI, USA; [7]David Geffen School of Medicine at University of California, Los Angeles (UCLA), Los Angeles, CA, USA, and the Lundquist Institute for Biomedical Innovation at Harbor-UCLA Medical Center, Torrance, CA, USA.

[†]These two authors contributed equally to this work.

[‡]These two authors contributed equally to this work.

[§]See appendix Supplementary Resource 1 for list of The Guthy-Jackson Charitable Foundation CIRCLES Study Group Co-Investigators

Supplementary Resource 1: Co-Investigators/Affiliated Co-Authors

Extramural Members of The Guthy-Jackson Charitable Foundation CIRCLES Study Group: Jeffrey L. Bennett, MD, PhD, Departments of Neurology and Ophthalmology, University of Colorado School of Medicine, Aurora, CO, USA. Participated in data collection, analysis interpretation and review of the manuscript. Eric C. Klawiter, MD, Department of Neurology, Massachusetts General Hospital, Harvard Medical School, Boston, MA, USA. Participated in data collection, analysis interpretation and review of the manuscript. Michael Levy, MD, PhD, Department of Neurology, Massachusetts General Hospital, Harvard Medical School, Boston, MA, USA. Participated in data collection, analysis interpretation and review of the manuscript. Sarah M. Planchon, PhD, Mellen Center for MS Treatment and Research, Neurological Institute, Cleveland Clinic, Cleveland, OH, USA. Participated in data collection, analysis interpretation and review of the manuscript. Pavle Repovic, MD, PhD, Swedish Medical Center, Seattle, WA, USA. Participated in data collection, analysis interpretation and review of the manuscript. Claire S. Riley, MD, Department of Neurology, Columbia University Medical Center, New York, NY, USA. Participated in data collection, analysis interpretation and review of the manuscript. Nancy L. Sicotte, MD, Cedars-Sinai Medical Center, Los Angeles, CA, USA. Participated in data collection, analysis interpretation and review of the manuscript. Ben Thrower, MD, Shepherd Center, Atlanta, GA, USA. Participated in data collection, analysis interpretation and review of the manuscript. Anthony Traboulsee, MD, Department of Medicine & Neurology, University of British Columbia, Vancouver BC, Canada. Participated in data collection, analysis interpretation and review of the manuscript.

Supplementary Resource 2. Summary of CIRCLES patient study file

		Cohort	
**Event**	**Form**	**Case**	**Control**
Enrollment	Inclusion/Exclusion	X	X
	Demographic Information	X	X
	Control Characteristics		X
Baseline	Case Baseline Encounter Summary	X	
	Control Baseline Encounter Summary		X
	Baseline Environmental Exposures Summary	X	X
	Baseline Activities of Daily Living	X	X
	Baseline Autoimmune Diseases	X	X
	Baseline Medications and Treatments	X	X
	Baseline Surgical and Medical Procedures	X	X
	Baseline Vaccinations	X	X
	Current Most Likely Diagnosis	X	
Follow-up	Case Follow-up Encounter Summary	X	
	Control Follow-up Encounter Summary		X
	Follow-up Environmental Exposures Summary	X	X
	Follow-up Activities of Daily Living	X	X
	Current Most Likely Diagnosis	X	

Supplementary Resource 3. Neurological examination for CIRCLES

**Prospective Forms**
Documentation from clinical record, ROS, vitals & interval history
Make sure clinical records for visits includes:
ROS – List std. general/other vomiting hiccups (yes/no)
Std vital signs
Interval symptoms – vision, motor, spinal cord, brainstem, cognition

**Condensed Neurological Examination**
Standard Neurological Examination specialized below
With special attention to Visual Acuity
25 ft walk: Record ambulation time
Kurtzke scale

**Details of Neurological Examination**			
All CNS (I-XII):	N	Abn	NT
Visual Acuity:	Snellen Chart Visualized – OS & OD		
	Worse than 20/400<br>20/400 (6/120)<br>20/200 (6/60)<br>20/100 (6/30)<br>20/70 (6/21)<br>20/50 (6/15)<br>20/40 (6/12)<br>20/30 (6/9)<br>20/25 (6/7.5)<br>20/20 (6/6)<br>20/15 (6/4.5)<br>Not documented<br>If not visualized – CF or HM or LP only – Blind (NLP)		

Motor – strength all 4 exts. / by std. scale 0-5/exts.
Coordination – cerebellar ataxia on either or both sides
DTR absent to clonus 0-4 std. scale – upper & lower ext. – all sites
Other reflexes required – Hoffman & Babinski

Sensory – all extremities mobility, pinprick, superficial touch, temp (document sensory levels)
Vibration (level of impairment)
Proprioception (level of impairment) – Each level (upper extremity) Finger, Wrist, Elbow
(lower extremity) Toe, Ankle, Knee
Bowel/Bladder function – Normal, Mild, Severe Include: Symptom/Catheter

Neurological Episode for CIRCLES

Date of Onset:	Date	
1st episode:	Y/N	
Recovery:	Complete/Incomplete/No Recovery	
Duration in days:	Duration (number of days)/ Ongoing/Unknown	
Dx of NMOSD:	New/Established/undetermined/If No-Specify	
TM:	Extensive/Non-Extensive	
ON:	OD	
	OS	
	Both	
	Chiasmal	
Brain Syndromes:	Brainstem	Cerebral Hemisphere

**Episode Symptoms:**	**Present: Y/N**	**New/Worsening/No Change**
Anorexia		
Mental deterioration		
Narcolepsy		
Psychiatric symptoms		
Hypothalamic dysfunction		
Visual acuity		
Facial motor abnormal		
Facial sensory abnormal		
Hiccups (recalcitrant)		
Hypoacusia		
Nausea		
Oculomotor impairment		
Speech/swallowing impairment		
Vertigo		
Vomiting (persistent)		
Bladder/bowel dysfunction		
Lhermitte’s sign		
Lower extremity dysfunction		

Sensory symptoms (e.g. pain, paresthesia)
Sexual dysfunction
Upper extremity dysfunction
Walking difficulties
Fatigue/weakness
Spasms
Other

Supplementary Resource 4: CIRCLES Baseline Activities of Daily Living

BASELINE ACTIVITIES OF DAILY LIVING FORM

Please note: Each page of the Baseline Activities of Daily Living Form contains instructions for completing any data fields that may be difficult or confusing. Please refer to the left hand side of the page for detailed data completion guidelines.

BASELINE ACTIVITIES OF DAILY LIVING FORM

General Form Instructions:

This form should be completed by interviewing the participant. This form is completed only once at baseline. Refer to the Follow-up Activities of Daily Living Form each time contact is made after the baseline encounter.

Question 1:

Please choose "Full-time" if the participant is able to do 8+ hours a day of school/work/housework activities.

Please choose "Part-time" if the participant is able to do less than 8 hours a day of school/work/housework activities.

Enter the date the last time the participant was able to attend school/work/do housework full-time. Date may be entered as a complete date (DD-MMM-YYYY) if known, or as a partial date if complete date is not known (MMM-YYYY or YYYY.)

If the day is unknown, check the "day unknown" box.
If the month is unknown, check the "month unknown" box.
Year must be provided for first episode onset and most recent episode.

Question 2:

Please choose "completely" if the participant is not able to care for themselves or live alone without significant assistance from another person.

Please choose "partially" if the participant can generally function by themselves with only occasional assistance from another person to complete day to day tasks.

BASELINE ACTIVITIES OF DAILY LIVING FORM

FORM PAGE 1 OF 2

Participant: Activities of Daily Living

Instrumental ADLs

1. **Is the participant able to attend school/work/do housework?**
   [ ] Yes
   [ ] No

   a. **If yes, specify:**
      [ ] Full-time
      [ ] Part-time

   b. **If part-time, when was the last time the participant was able to attend school/work/do housework full-time?**

      Date: _____-_____-________
      DD MMM YYYY

      Day unknown _____ Month unknown _____

2. **Is the participant dependent on others for care?**
   [ ] Completely
   [ ] Partially
   [ ] No

BASELINE ACTIVITIES OF DAILY LIVING FORM

General Form Instructions:

This form should be completed by interviewing the participant. This form is completed only once at baseline. See instruction on page 2.

Table:

Specify the participant's capabilities to perform the basic ADLs.

Level of ability to perform for Bathing/Showering through Personal Grooming:
Choose "Independent" if the participant never requires assistance to complete this task.
Choose "Partially Dependent" if the participant requires assistance part of the time to complete this task.
Choose "Completely Dependent" if the participant always requires assistance to complete this task.

Level of ability to perform for Mobility:
Choose "Independent" if the participant never requires assistance to complete this task.
Choose "Partially Dependent" if the participant requires assistance part of the time to complete this task.
Choose "Completely Dependent" if the participant always requires assistance to complete this task.
Choose "Bedbound" if the participant is unable to use any assisted devices.

Bedbound – Confined or unable to leave one's bed because of infirmity, weakness or illness.

Question 3:

Question 3 is answered only if the Mobility level in the ADL table is answered as 'partially dependent'.

If "Yes", please document which type of mobility assistance device is used.

One point assistance includes the use of a cane, rail, or another person (unilateral assistance.)
Two point assistance includes the use of two canes, or a walker (bilateral assistance). If using a cane and a person, this should be marked as two point assistance.

Question 4:

Question 4 is answered only if the Mobility level in the Basic ADLs table is answered as 'completely dependent.'

If "Yes," please document which type of mobility assistance device is used.

One point assistance includes the use of a cane, rail, or another person (unilateral assistance.)
Two point assistance includes the use of two canes, or a walker (bilateral assistance.) If using a cane and a person, this should be marked as two point assistance.

BASELINE ACTIVITIES OF DAILY LIVING FORM

FORM PAGE 2 OF 2

Basic ADLs

2a.

Activity	Level of ability to perform
Bathing/Showering	[ ] Independent<br>[ ] Partially Dependent<br>[ ] Completely Dependent
Bowel/Bladder Management	[ ] Independent<br>[ ] Partially Dependent<br>[ ] Completely Dependent
Food Preparation	[ ] Independent<br>[ ] Partially Dependent<br>[ ] Completely Dependent
Eating	[ ] Independent<br>[ ] Partially Dependent<br>[ ] Completely Dependent
Dressing	[ ] Independent<br>[ ] Partially Dependent<br>[ ] Completely Dependent
Personal Grooming	[ ] Independent<br>[ ] Partially Dependent<br>[ ] Completely Dependent
Mobility	[ ] Independent<br>[ ] Partially Dependent<br>[ ] Completely Dependent<br>[ ] Bedbound

3. **If mobility level above is marked "Partially Dependent," are any assistance devices used?**
   [ ] Yes
   [ ] No

   a. **If yes, specify (check all that apply):**
      [ ] One point assistance
      [ ] Two point assistance
      [ ] Wheelchair

4. **If mobility level above is marked "Completely Dependent," are any assistance devices used?**
   [ ] Yes
   [ ] No

   a. **If yes, specify (check all that apply):**
      [ ] One point assistance
      [ ] Two point assistance
      [ ] Wheelchair
